# The prevalence, awareness, and control of hypertension among workers in West Africa: a systematic review

**DOI:** 10.3402/gha.v8.26227

**Published:** 2015-01-22

**Authors:** William K. Bosu

**Affiliations:** Department of Epidemics and Disease Control, West African Health Organisation, Bobo-Dioulasso, Burkina Faso

**Keywords:** West Africa, hypertension, blood pressure, awareness, treatment, control, workers, systematic review

## Abstract

**Background:**

Interventions in workplace settings are considered to be cost-effective in preventing cardiovascular diseases. A systematic review was conducted to assess the prevalence of hypertension and the level of awareness and control among workers in West Africa.

**Design:**

A systematic search for studies on formal and informal sector workers aged ≥15 years in West Africa published between 1980 and September 2014 was undertaken using the Ovid Medline, Embase, PubMed, and Google Scholar databases. Clinical and obstetric studies and studies that did not report prevalence were excluded. Data on study settings, characteristics of workers, blood pressure (BP) levels, prevalence of hypertension, and associated demographic factors were extracted.

**Results:**

A total of 45 studies from six countries were identified involving 30,727 formal and informal sector workers. In 40 studies with a common definition of hypertension, the prevalence ranged from 12.0% among automobile garage workers to 68.9% among traditional chiefs. In 15 of these studies, the prevalence exceeded 30%. Typically sedentary workers such as traders, bank workers, civil servants, and chiefs were at high risk. Among health care workers, the prevalence ranged from 17.5 to 37.5%. The prevalence increased with age and was higher among males and workers with higher socio-economic status. Complications of hypertension, co-morbidities, and clustering of risk factors were common. The crude prevalence of hypertension increased progressively from 12.9% in studies published in the 1980s to 34.4% in those published in 2010–2014. The proportion of hypertensives who were previously aware of their diagnosis, were on treatment or had their BP controlled was 19.6–84.0%, 0–79.2%, and 0–12.7%, respectively. Hypertensive subjects, including health workers, rarely checked their BP except when they were ill.

**Conclusions:**

There is a high prevalence of hypertension among West Africa's workforce, of which a significant proportion is undiagnosed, severe or complicated. The clustering of risk factors, co-morbidities, and general low awareness warrant an integrated and multisectoral approach. Models for workplace health programmes aiming to improve cardiovascular health should be extended to informal sector workers.

Two of the earliest post-colonial studies on the cardiovascular health of workers in West Africa were described in Nigeria and in Ghana more than 40 years ago. In Nigeria, Akinkugbe and Ojo assessed arterial blood pressure (BP) levels among 821 workers in a tobacco factory and a shopping centre in Ibadan in 1968 (1). In Ghana, Pobee et al. screened 6,900 civil servants in 1973 as part of a World Health Organization Feasibility Study on the Community Control of Hypertension (2). Since these publications, there has been a rapid rise in cardiovascular diseases (CVDs) and non-communicable diseases (NCDs) in West Africa (3, 4). According to the Global Burden of Disease (GBD), the ranking of hypertension worsened from the fourth to the third leading risk factor for deaths in West Africa from 1990 to 2010 (5). Cardiovascular complications are more common and severe in sub-Saharan Africa, and they occur at younger ages (6, 7). With the increasing urbanisation, globalisation, and associated nutrition transition, NCDs are likely to increase in the region (8–11).

However, the development of occupational health services has not kept pace with this rapid rise in most West African countries. This is in spite of the determination that workplace health programmes are among the most cost-effective ways to prevent NCDs (12). The United Nations Political Declaration on NCDs also calls on the private sector to create ‘an enabling environment for healthy behaviour among workers’ and promote ‘safe and healthy working environment’ (13). A major challenge is that most workers in West Africa are engaged in the informal sector without access to any structured occupational health programme to protect or promote their health. Even among the formal sector workers who should have access to pre-employment screening and periodic medical screening, awareness of hypertension is low with consequent low control rates and high levels of target organ damage (14).

Insufficient attention has been paid to the prevention and control of NCDs in West Africa, particularly programmes that target formal and informal sector workers. Data are required to guide evidence-informed decisions and to advocate for change. However, there is a dearth of studies on hypertension at the sub-regional level in West Africa. The few regional studies on the prevalence of hypertension have been undertaken as part of reviews in Africa as a whole (15, 16) or limited to a few countries in the sub-region (17, 18). Review studies on hypertension among workers and identifiable social groups in West Africa are notably absent. The present study was therefore undertaken to review the prevalence of the hypertension among workers in the Economic Community of West African States (ECOWAS). The review assessed the trends and severity of hypertension as well as the knowledge, awareness, treatment practices, and extent of control among the workers. It responds to the resolution of the 11th Assembly of the ECOWAS Ministers of Health in 2010 which calls for a higher priority to the prevention and control of NCDs and for improved surveillance (19).

## Methods

### Study area

ECOWAS was established in 1975 with the aim of fostering regional political and economic integration among 15 West African Member States. The Member States comprise eight Francophone countries (Benin, Burkina Faso, Cote d'Ivoire, Guinea, Mali, Niger, Senegal, Togo), five Anglophone countries (The Gambia, Ghana, Liberia, Nigeria, Sierra Leone), and two Lusophone countries (Cape Verde, Guinea-Bissau). The 15 Member States have a combined population of about 320 million, accounting for about 43% of the sub-Saharan African (SSA) population. There has been rapid urbanisation with 42% living in urban areas in 2010, a two-fold increase over the 19% 40 years earlier. The population living in urban areas is projected to reach 63% by 2050 (20).

Poverty in West Africa is among the worst in the world with about 30–45% of the population in most countries living under $1.25 purchasing parity power (PPP) per day (21). Eleven of the ECOWAS Member States are in the bottom 30 of the Human Development Index league of 185 countries. The life expectancy at birth is about 54 years, ranging from 45 years in Sierra Leone to 75 years in Cape Verde (22). The major health problems include malaria, diarrhoeal diseases, acute respiratory infections, undernutrition, HIV, and hypertensive diseases. There are periodic outbreaks of cholera, meningitis, yellow fever, and Lassa fever.

While much of West Africa is in the early stages of the nutrition transition, three countries – Ghana, Cape Verde, and Senegal – are in the later stages (23). In many West African cities, more than 25% of adults have hypertension with rates higher than 40% being reported in Ouagadougou, Accra, and St. Louis (24–26) . The high prevalence of hypertension is not limited to the affluent populations. Among urban poor adults in Ouagadougou and Accra, 19 and 28% have hypertension (27, 28). A recent review of diabetes in Africa reported prevalence ranging from 2.5 to 7.9% (29). Among some workers in Accra and Dakar, the prevalence exceeds 9.0% (30, 31). Obesity rates in West Africa increased by about 115% to reach 15% in the 15-year period from 1990 to 2004 (9). Risk factors for NCDs are evident in children and adolescents in Africa, about 10.6% of whom are overweight/obese (32). The proportion of West Africans not engaged in vigorous physical activity varies from 31.0% in Sierra Leone to 93.0% in Cote d'Ivoire (33).

### Search strategy and data extraction

A literature search was conducted on the Ovid Medline, Embase, and PubMed databases using the search terms for hypertension, Africa, and workers in a systematic build up. In the combined Ovid Medline and Embase databases, the terms ‘hypertension’ and ‘occupation’ were exploded while in PubMed, the Medical Subject Headings (MeSH) ‘sex workers’, ‘social work’, ‘occupations’, ‘occupational groups’, ‘health personnel’, ‘community health workers’, ‘agriculture’, and ‘manpower’ as well as ‘hypertension’ were used. In each database, these headings were complemented by a long list of generic terms for workers (artisans, company, corporation, employees, employers, enterprise, occupation, ‘occupational groups’, profession, staff, ‘blue collar’, ‘green collar’, ‘pink collar’, ‘white collar’, workers, workplace). In order to capture work types that are peculiar to the African setting, more specific search terms (agriculture, ‘agricultural workers’, ‘bank workers’, chiefs, ‘civil servants’, executive, factory, ‘factory workers’, farmers, ‘health workers’, industry, ‘industrial workers’, laborer, labourer, lecturers, market, plantation, teachers, traders, tradesmen, trading) were also added.

For the disease, the subject headings were complemented by a search for articles whose title or abstract contained the terms ‘hypertension’ or ‘blood pressure’. For the geographical scope, Africa and the names of each of the 15 individual ECOWAS Member States were used. The various search terms were connected with the relevant Boolean operators. The Ovid Medline database search also included a search on related terms. Additional searches were also done in Google Scholar in French and in English. Manual searches of listed references were done to maximise the number of identified studies. Duplicate records were removed using the EndNote reference manager (34). Then the titles were reviewed, and articles obviously not related to the subject of interest were removed. The abstracts or full text of the remaining articles was then reviewed, and further articles were removed if they did not fulfil the inclusion criteria. Articles published between 1980 and September 2014 were retrieved based on the consideration that 35 years was long enough duration to allow patterns and trends in hypertension among workers to be determined.

Studies were included if they were conducted among formal or informal workers aged ≥15 years, the sample size was ≥75 workers, and they reported an estimate of the prevalence of hypertension. Studies conducted among patients or the general population, those that were obstetric, pharmacological, anthropological, review studies, or studies outside the ECOWAS region were excluded. Studies involving pre-selection medical examination for possible recruitment into jobs were also excluded. Multiple studies among the same set of workers were counted once, but the separate papers reporting the prevalence of hypertension were included in the references.

Using a standard data extraction sheet, data on the study location, type of setting, study population, sampling technique, BP measurement technique, mean age, sex distribution, prevalence of overweight or obesity, comorbidities, mean systolic blood pressure (SBP) and diastolic blood pressures (DBP), prevalence of diabetes mellitus, and the prevalence of hypertension were obtained. Most of the studies used the threshold 140/90 mmHg and/or taking of antihypertensive medication for their definition of hypertension, in line with the Seventh Report of the Joint National Committee on the Prevention, Detection, Evaluation and Treatment of High Blood Pressure (JNC VII) (35). A few studies used the older 160/95 mmHg cut-off point in line with existing WHO guidelines (36).

Besides the prevalence of hypertension, the severity of hypertension was assessed by the prevalence of grade 2 (SBP of 160–179 mm Hg or DBP of 100–109 mmHg) or grade 3 hypertension (SBP of ≥180 mm Hg or DBP was ≥110 mmHg), history of hospitalisation, and the presence of complications and co-morbidities (35). The proportion of hypertensive patients who reported having previously been diagnosed by a health professional was considered to be aware of their diagnosis. The proportion of persons with hypertension on treatment was based on the subjects reporting taking antihypertensive medication. The proportion of those who were aware of their diagnosis and who were on antihypertensive therapy was also calculated where available. BP was deemed to be under control if it was less than 140/90 mmHg in subjects on medication. No pooled analyses could be performed in view of the heterogeneity of the study population.

## Results

### Description of the studies

**Fig. 1:**
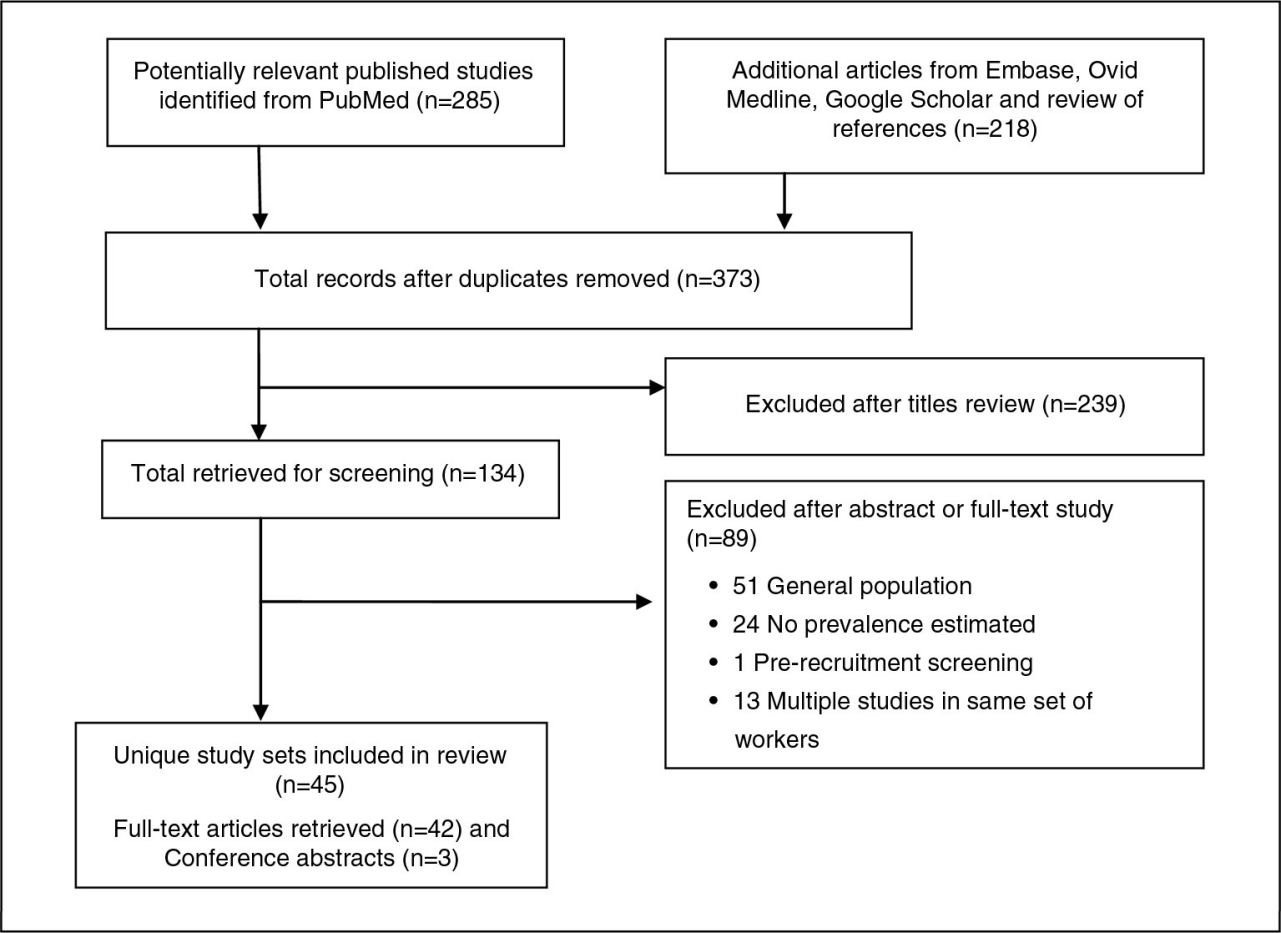
The process of selecting articles.

**Fig. 2:**
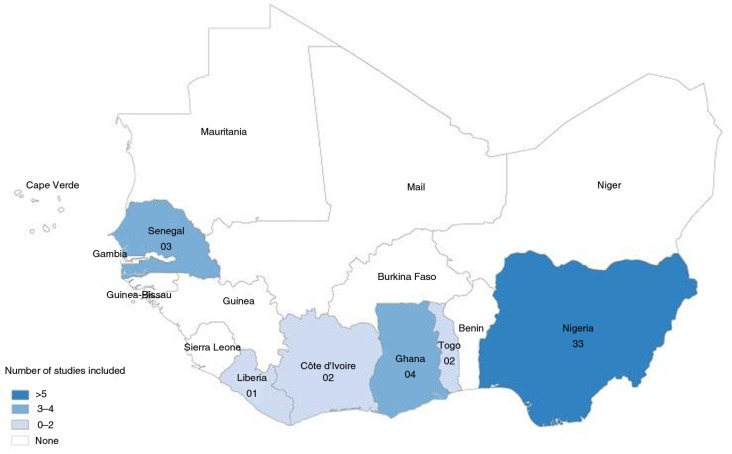
Map of West Africa showing distribution of selected studies.

A total of 45 papers on hypertension were identified from six countries – Cote d'Ivoire, Ghana, Liberia, Nigeria, Senegal, and Togo (Fig. 1). Thirty-three (73.3%) of them were from Nigeria, four from Ghana, three from Senegal, two each from Cote d'Ivoire and Togo, and one from Liberia (Table 1 and Fig. 2). Five of the papers were published in French. The studies covered a wide range of workers including largely sedentary groups such as market women (37–40) or long-distance drivers (41) and largely active groups such as farmers (42, 43) or factory workers (44–46) .

**Table 1:** Characteristics of studies on prevalence of hypertension among workers

No.	Country	References	Study population	Location	Setting	Year of study	Sample size	Participation rate	Sampling representativeness	% Female	Age group (years)	Mean age±sd
1	Cote d'Ivoire	Konin et al. (64)	Health workers	Abidjan	Urban		821		Probable	59.4	28–58	42.9
2	Cote d'Ivoire	Koffi et al. (76)	Port workers	Abidjan	Urban	1995	202		Yes	13.4	30–55	46.0±6.0
3	Ghana	Addo et al. (30)	Civil servants	Accra	Urban	2006	1,015	82.7	Yes	39.4	25–68	44.0±10.1
4	Ghana	Gunga et al. (78)	Goldminers and rubber company workers	Tropical rainforest	Urban	1986, 1988	495		Yes			
5	Ghana	Amidu et al. (71)	Male automobile garage workers	Kumasi	Urban	January–March 2009	200		No/ND	0.0		30.2±7.8
6	Ghana	Aryeetey & Ansong (60)	University staff	Accra	Urban	June 2009	141	99.3	Yes	32.6		40.5±10.8
7	Liberia	Giles et al. (43)	Rubber plantation workers		Rural	September–November 1989	3,588	83.5	Yes	44.3	20–55+years	
8	Nigeria	Abidoye et al. (75)	Airport Authority workers	Lagos	Urban	July–August 2000	380		Yes	37.1		
9	Nigeria	Kadiri et al. (73)	Bank workers	Ibadan	Urban		917	>95	Yes	33.7	18–64	Men 34.3±7.7; women 32.3±7.0
10	Nigeria	Abidoye et al. (74)	Bank workers	Lagos	Urban		530		Yes		20–59	
11	Nigeria	Bunker et al. (79)	Civil servants	Sokoto	Urban	Summer 1990	539		Probable	13.5	20–54	
12	Nigeria	Huston et al. (51)	Civil servants	Benin City, Edo State	Urban	1992	766	84.3	Yes	37.1	20–64	41
13	Nigeria	Olatunbosun et al. (53)	Civil servants	Ibadan	Urban		998		Yes	41.8	19–70	40.0±8.3
14	Nigeria	Oyeyemi & Adeyemi (55)	Civil servants and health workers	Maiduguri	Urban		292	79.8	Yes	34.9	20–65	44.8±8.5
15	Nigeria	Bunker et al. (47); Bunker et al. (48)	Civil servants from State Ministries	Bendel State	Urban	1987 and 1988	559		Probable	21.6	25–54	Men 37.8; women 34.9
16	Nigeria	Ekpo et al. (50)	Civil servants, factory and plantation workers	Calabar, Cross River State	Urban		5,200	98	Yes	15.7	16 years and above	
17	Nigeria	Olugbile & Oyemade (42)	Farmers and industrial workers	Badeku, Ewereko villages	Rural		276	78.9	Yes		21–70	97.8% of industrial workers and 55.7% of farmers <50 years
18	Nigeria	Balogun & Owoaje (37)	Female traders	Sango Market, Ibadan, Oyo State	Urban	April 2003	281		Yes	100		37.3±12.8
19	Nigeria	Odugbemi et al. (39)	Female traders	Tejuosho market, Lagos	Urban	August–September 2006	400		Yes	100		Men 45.5±11.9; women 42.3±11.0
20	Nigeria	Uwanuruochi et al. (80)	Health care workers	Umuahia, Abia State	Urban	October 2010	299		No/ND	72.6	40–60	47.7±5.4
21	Nigeria	Funke & Ibrahim (62)	Health workers	Jos City, Plateau State	Urban	June–September 2005	340	100	Yes	62.9	24–60	
22	Nigeria	Owolabi et al. (65)	Health workers	Ogbomoso, Oyo State	Urban		324	92.3	Yes	55.9	20–65	41.1±10.1
23	Nigeria	Adeoye et al. (66)	Health workers in a tertiary hospital		Urban		352		Probable	63.6		42.03±9.4
24	Nigeria	Ogunlesi et al. (44)	Male battery factory workers	Ibadan	Urban	November 1989	404	100	Yes	0.0	18–54	
25	Nigeria	Ofuya (81)	Male commercial motorcyclists; market women	Port Harcourt, Rivers State	Urban		200		Yes		*M*=16–56 years; *F*=16–54 years	Men=23.1; women=25.0
26	Nigeria	Oviasu & Okupa (54); Oviasu & Okupa (82)	Male office clerks, male field labourers (rural); civil servants (urban)	Isiuwa village and Benin City, Bendel State	Rural and Urban	Rural=June–July 1976; Urban=September–November 1977	1,263	Rural male 98.8; Urban 95.0	Yes	Rural 0.0; Urban 27.5	15–60	
27a	Nigeria	Kaufman et al. (70)	Male rural farmers	Idere village, Ibarapa district, Oyo State	Rural	1994	108		Yes	0.0	45 years and above	63.0±12.6
27b	Nigeria	Kaufman et al. (70)	Retired railway workmen	Ibadan	Urban	1994	203			0.0	45 years and above	60.2±8.5
28	Nigeria	Ulasi et al. (40)	Market workers	Ogbete market, Enugu State	Urban	May 2006	688		No/ND	48.5		38.0±13.3
29	Nigeria	Ordinioha (59)	Medical school lecturers	Port Harcourt	Urban		75	75	Yes	34.7		46.1±9.6
30	Nigeria	Ebare et al. (83)	Musical shop operators	Benin City, Edo State	Urban		250	83.3	Yes	12.0		26.9±7.6
31	Nigeria	Shittu et al. (46)	Pharmaceutical industry workers		Urban		750		Yes	58.0		
32	Nigeria	Idahosa (52)^a^	Policemen and male civil servants	Benin City, Bendel State	Urban	May 1983	1,115		No/ND	0.0	Policemen 20–63; civil servants 20–62 years	Policemen 23.2 years; civil servants 28.6 years
33	Nigeria	Amoran et al. (41)	Professional drivers	Sagamu Local Government Area, Ogun State	Urban	February–March 2008	400		Probable	0	21–62	41.1±6.1
34	Nigeria	Okojie et al. (68)	Senior executives of industries and companies	Benin City, Edo State	Urban		202		Yes	23.3	25–64	
35	Nigeria	Oghabon et al. (67)	Staff of a government organisation and private industry	Illorin	Urban		281		Probable	24.9		40.3±9.6
36	Nigeria	Charles-Davies et al. (38)	Traders	Bodija market, Ibadan	Urban		534		Yes	68.2	18–105	
37	Nigeria	Ordinioha & Brisibe (77)	Traditional chiefs	Rivers State	Urban; semi-urban		106		Yes	0.0		56.5±4.1
38	Nigeria	Emerole et al. (57)	University staff	Owerri, Imo State	Urban	October 2003	241		Yes	49.4		
39	Nigeria	Ige et al. (58)	University staff	Ibadan	Urban		525	96.0	Yes	48.8		37.4±9.5
40	Nigeria	Omokhodion & Kolude (72)	Vegetable, cereal and tuber mill operators	Bodija market, Ibadan	Urban		120		No/ND	45.8	18–65	41
41	Senegal	Lang et al. (45)	Factory and hotel workers	Dakar	Urban		1,869	97	Yes	29.6	16–64	Men 39.3±9.7; women 35.4±8.8
42	Senegal	Seck et al. (31)	Information technology workers	Dakar	Urban	September– November 2010	402	100	Yes	33.8		46.2±7.6
43	Senegal	Mbaye et al. (69)	Telecommunication workers	Not stated	Urban	2006	1,229		Probable	29.8	21–58	41.8±9.1
44	Togo	Atatsi et al. (61)	University staff	Lome	Urban	May–June 2006	640		No/ND	36.1	21–60	Men=41; women=43
45	Togo	Yayehd et al. (56)	Civil servants	Lome	Urban	June 2010	207	97.6	Yes	32.9	24–60	42.7±9.8

a Benin City was located in Bendel State but the State was divided up into Edo State and Delta State in 1991, with the capital remaining in the former. SD=standard deviation; ND=not determined.

The most commonly studied groups were civil servants in whom there were 10 studies in three countries, Ghana, Nigeria, and Togo (30, 47–56) . The other workers were university staff (57–61) , health care workers (62–66) , office workers (31, 67–69) , policemen (52), artisans (70–72) , bank workers (73, 74), civil aviation workers (75), port workers (76), traditional chiefs (77) and miners (78). Eight studies involved exclusively male workers (41, 44, 52, 70, 71, 77) or female workers (37, 39). Traders and health workers studied were predominantly female while automobile garage workers, drivers, railway workers, policemen, factory workers, civil servants, and chiefs were predominantly male (Table 1). Except for two studies conducted in rural populations (42, 43), the studies were conducted in urban or mixed settings.

Thirty-two studies (80.0%) were considered to have used an unbiased sampling technique or recruited their entire workforce while a further seven studies (15.5%) provided evidence of high participation (Table 1). Representative samples from formal workplaces with staff lists such as airport authority (75), banks (73, 74), civil service ministries (51, 53, 55, 82, 84), hospitals (65), and universities (57, 59) were obtained through simple random, systematic, cluster, or stratified sampling. Representative samples were also obtained in studies with informal sector workers such as port workers (76), factory workers (44–46) , music shop operators (83), hotel workers (45), traders (37–39) , and farmers (43, 70) through the use of registers or a sampling of their mapped locations (39, 43). Representativeness was easily obtained in small to medium-sized institutions through the enrolment of all their workers. For example, in the study with the smallest sample size in this review, a total of 75 (68.8%) lecturers (based on a calculated sample size) were randomly sampled from a total of 109 eligible medical school lecturers in Port Harcourt, Nigeria (59).

Some studies that did not report representative sampling obtained high participation of their workers. For example, investigators assessing hypertension in civil servants in two different studies in separate locations could not obtain any staff lists as these were not available nor did they consider it feasible to prepare one (48, 79). Consequently, they achieved high participation through a door-to-door mobilisation of all on-site workers. Without a sampling frame of all eligible workers, they could not provide any response rates based on eligible subjects.

Incomplete reporting did not permit the assessment of representativeness of workers in a few studies. For example, a study among long-distance professional drivers estimated the needed sample size but did not provide adequate information on the sampling procedure (41). Studies among automobile garage industry workers (71), health care workers (80), policemen (52) or mill operators (72) were compelled to recruit a convenient sample of workers as a sampling frame was unavailable, and it was not feasible to compile one. In such cases, recruitment was facilitated through the solicited cooperation of the leader of these categories of workers.

The maximum sample size of 5,200 involved a mixed group of civil servants and factory and plantation workers in South-eastern Nigeria (50). Overall, the mean and total sample size in all 45 studies was about 683 and 30,727 workers, respectively. Reported participation rates ranged from 75 to 100%. The age of the workers ranged from 15 years old civil servants (85) to 105 years old traders (38). In 32 reporting studies, the mean ages ranged from 23.2 years among policemen (52) to 63.0±12.6 years among rural farmers (70). Overall, most workers studied were in their 40s, and the youngest workers were in their 20s (52, 83).

Of the 45 studies, three were published in the 1980–1989 decade, 10 in 1990–1999, 12 in 2000–2009, and 20 from 2010 to September 2014. The annual rate of production therefore increased progressively from 0.3 in the 1980s through to 1.2 in the 2000s and then sharply to 4.2 in 2010–2014.

### BP measurement

Strategies to improve the quality of BP measurements included the use of trained personnel or the self-deployment of the study researchers in the process, the use of formally-certified field workers, ensuring that the subject was relaxed and seated upright with legs uncrossed and flat on the floor with the arm supported at the level of the heart, the use of calibrated equipment with appropriate cuff size and the use of multiple measurements.

There was a wide variation in the quality of the reported approaches to the measurement of BP (Table 2). Of the 35 studies which provided information, 29 measured the BP during a single visit. In a few studies, subjects with raised BP were advised to make one or two additional visits for further measurements (30, 43, 64). Other studies required two or three visits for all subjects for BP measurements (51, 79). The interval between visits ranged from 1 day to 3 weeks. Most studies took two or three BP measurements and used the mean of at least two of them for analysis. Of 12 studies which took three BP readings, six used the mean of the latter two readings for analysis. Four studies measured the BP only once or used only one reading (after rejecting the first reading) in their analyses (50, 53, 54, 81). One-third of the studies reviewed did not provide adequate information on how the BP was measured.

**Table 2:** Blood pressure (BP) measurement techniques in studies among workers in West Africa

No.	References	Personnel taking BP	No. of visits	Interval between visits	Frequency of readings per visit	Initial rest time (min)	Interval between multiple readings (mins)	Reading used in analysis	cuff size	Posture	Body part for cuff	Device
1	Konin et al. (64)		1–3. Those with raised BP on 1st visit and known hypertensives made 2 additional visits			10–15						Manual
2	Koffi et al. (76)		1		2					Supine		
3	Addo et al. (30)	Trained interviewers	1–2, BP repeated at a later visit if initially >140/90 mmHg without treatment	3 weeks	3	≥10	1	Mean of 2nd & 3rd readings	Appropriate cuff size	Seated	Right arm	Electronic – Omron M51
4	Gunga et al. (78)											
5	Amidu et al. (71)	Qualified nurses	1		2	≥5	5	Mean of 2 readings			Left arm	Manual
6	Aryeetey & Ansong (60)	Trained personnel	1		2	5	10	Mean of 2 readings			Left arm	Manual – Accoson MK.3
7	Giles et al. (43)		1–2, 2nd visit for those with raised BP at 1st visit		2 readings at 1st visit, 3 readings at 2nd visit	5		Mean of 3 readings			Left arm	Electronic – Dynamap 8100
8	Abidoye et al. (75)											
9	Kadiri et al. (73)	Trained physicians	1		3		1	Mean of 3 readings	Appropriate cuff sizes			Manual
10	Abidoye et al. (74)									Seated		Manual
11	Bunker et al. (79)	Trained observers	2		1	≥5		Mean of the 2 readings from each visit	Appropriate cuff size	Seated	Arm	Manual – Baumanometer
12	Huston et al. (51)	Certified technicians	3	2 days after 1st visit and 1 day after 2nd visit	3	5	1	Mean of latter 2 readings over all the three visits			Arm	Manual
13	Olatunbosun et al. (53)		1		1			Single reading			Left arm	Manual
14	Oyeyemi & Adeyemi (55)		1		3	5	3–5	Mean of 3 readings			Left arm	Electronic – Dinamap 8100/8101
15	Bunker et al. (47)	Certified medical students	1		3	≥5		Mean of latter 2 readings	Appropriate cuff size	Seated	Arm	Manual – Baumanometer
16	Ekpo et al. (50)	Trained observers	1		2	≥5		Only the 2nd reading analysed	Appropriate cuff size		Arm	Manual
17	Olugbile & Oyemade (42)											
18	Balogun & Owoaje (37)		1		2	5	5		Appropriate cuff size	Seated	Right arm	Electronic – OMRON
19	Odugbemi et al. (39)	Trained observers	1		2		2	Mean of 2 readings		Seated	Right arm	Manual
20	Uwanuruochi et al. (80)	Physicians	1						12×25 cm			Manual – Accoson
21	Funke & Ibrahim (62)	Physicians	1		3	≥3		Mean of 3 readings	Appropriate cuff size	Seated	First 2 measured on left arm and 3rd on right arm	Manual
22	Owolabi et al. (65)		1		2	5	5	Mean of 2 readings	Cuff size 12.5 cm	Seated	Left arm	
23	Adeoye et al. (66)											Manual – Accoson
24	Ogunlesi et al. (44)		1		3	5		Mean of 3 readings	One of three cuff sizes used as appropriate		Right arm	Electronic
25	Ofuya (81)		1		1	≥5		Single reading		Seated	arM	Electronic – OMRON Hem-412C
26	Oviasu & Okupa (54)		1		1	5		Single reading	12×22 cm	Seated	Left arm	Manual
27	Kaufman et al. (70)	Trained observers	1		3	5–10			Appropriate cuff sizes			Manual
28	Ulasi et al. (40)		1		3	10	5	Mean of 3 readings	Appropriate cuff size		Non-dominant arm	Manual – Accoson
29	Ordinioha (59)		1		3		≥3	Mean of latter 2 readings	Appropriate cuff size	Seated	Arm	Manual
30	Ebare et al. (83)		1		2		15	Mean of 2 readings		Seated	Right arm	Manual
31	Shittu et al. (46)											
32	Idahosa (52)	Two staff nurses who cross-checked unusual BP readings	1		2	≥5		Mean of 2 readings	14×52 cm	Seated	Right arm	Electronic – UEADA 8000 validated against Accoson mercury device
33	Amoran et al. (41)	Trained health workers										
34	Okojie et al. (68)		1		2			Mean of 2 readings		Seated	Arm	Manual
35	Oghabon et al. (67)		1–2, subjects with raised BP re-evaluated	1 day	2	5–10				Seated	Both arms in same subject	Manual
36	Charles-Davies et al. (38)											
37	Ordinioha & Brisibe (77)		1		3			Mean of latter 2 readings	Appropriate cuff size	Seated	Arm	Manual
38	Emerole et al. (57)	Physicians	1									Manual
39	Ige et al. (58)											
40	Omokhodion & Kolude (72)		1									
41	Lang et al. (45)	Researchers	1		2		≥5	Mean of 2 readings		Seated		Manual
42	Seck et al. (31)	Skilled doctors and nurses	1									
43	Mbaye et al. (69)	Physicians	1		1–2, BP measurement repeated if 1st is raised		≥5	2nd measurement used in the analysis, if 1st measurement is raised		Seated		Electronic
44	Atatsi et al. (61)											
45	Yayehd et al. (56)		1		3		15	Mean of latter 2 readings		Seated	Both arms in the same subject	Manual

There were also variations in the device used for the BP measurement. Of the 33 studies reporting their measurement device, 25 employed a mercury sphygmomanometer while eight employed an electronic monitor. One study validated their electronic BP monitor using a manual sphygmomanometer (52). Only 25 studies reported the part of the body to which the cuff of the BP device was applied. BP was taken in both arms in the same subjects in three studies, on the left arm in seven studies, and on the right arm in six studies.

### Prevalence of hypertension

The prevalence of hypertension among the workers was generally high, the wide diversity of workers notwithstanding. In 40 studies which used the 140/90 mmHg threshold, the prevalence ranged from 12% among automobile garage workers in Ghana (71) to 69% among traditional chiefs in the oil-rich Ogba land in the Rivers State, Nigeria (77) (Table 3). These two extreme groups prevalence were quite different in terms of socio-demographic characteristics and risk factors. The artisans were younger (mean ages 30 years vs. 57 years), less affluent, less likely to be obese (2% vs. 26%), and less likely to drink alcohol (23% vs. 93%) or smoke (5% vs. 25%). Overweight or obese chiefs were 2.25 times as likely as those with normal weight to be hypertensive (78.3% vs. 34.8%).

**Table 3:** Mean systolic and DBPs and prevalence of hypertension among workers using the 140/90 and 160/95 mmHg BP cut-off points

			Prevalence BP 140/90 mmHg %			Prevalence BP 160/95 mmHg %				
				
No.	Study population	Mean age±sd	Males	Females	Total sample	Males	Females	Total sample	Mean SBP	Mean DBP
1	Airport authority workers (75)^a^				SHT=21.7; DHT=18.2			Severe SHT=3.9; severe DHT=0.7		
2	Bank workers (73)	Men 34.3±7.7; women 32.3±7.0	22.2	14.2	19.5	10.4	7.1	9.3	Men 118.2±17.3; women 113.7±16.7	Men 76.1±11.5; women 72.4±11.4
3	Bank workers (74)^b^				SHT=17.9; DHT=21.1					
4	Civil servants (30)^c^	44.0±10.1	31.7	28.0	30.2				128.5	79
5	Civil servants (79)		19.3	13.7	18.6	6.2	4.1	5.9	Men (20–54 years) 124.7±10.9; women (20–44 years) 121.5±15.6	Men (20–54 years) 73.4±10.9; women (20–44 years) 73.8±9.7
6	Civil servants (51)	41	19.3	9.9	15.8				117.4±17.6	76.1±13.5
7	Civil servants (53)^d^	40.0±8.3				13.9	5.3	10.3		
8	Civil servants and health workers (55)	44.8±8.5	20.5	25.5	23.3				129.2±16.8	81.3±10.5
9	Civil servants (47, 48)	Men 37.8; women 34.9	34.2	16.5	30.4	17.8	10.7	16.3	Men 127.9±17.7; women 116.4±15.1	Men 82.2±12.6; women 75.4±11.3
10	Civil servants, factory and plantation workers (50)^d^					8.9	3.5	8.1		
11	Civil servants (56)	42.7±9.8			54.1					
12	Factory and hotel workers (45)	Men 39.3±9.7; women 35.4±8.8	21.9	19.9	21.3	7.4	10.1	8.2	Men 126.7±17.5; women 123.3±20.2	Men 75.2±11.6; women 74.6±11.7
13	Farmers and industrial workers (42)^d^	97.8% of industrial workers and 55.7% of farmers <50 years			12.3					
14	Female traders (37)	37.3±12.8		19.9	19.9		6.8	6.8	122.0±20.0	78.0±13.0
15	Female traders (39)	Men 45.5±11.9; women 42.3±11.0		34.8	34.8					
16	Goldminers and rubber company workers (78)^d,e^							8.9		
17	Health workers (80)	47.7±5.4			37.5				128.0±16.8	80.5±10.5
18	Health workers (64)	42.9	17.7	17.4	17.5					
19	Health workers (62)				36.5					
20	Health workers (65)^d^	41.1±10.1			20.1					
21	Health workers (66)	42.03±9.4			34.9					
22	Male automobile garage workers (71)^d^	30.2±7.8	12.0		12.0				122.3±17.5	75.9±11.6
23	Male battery factory workers (44)		20.3		20.3	8.4		8.4	128.0±13.4	69.6±11.0
24	Male commercial motorcyclists; market women (81)	Men=23.1; women=25.0	16	12					Men 136; women 122	Men 81 women 83.4
25	Male civil servants, male field labourers (54, 82)^d,e^					14.4	10.4	13.3	Rural clerks 127.7±17.1; rural labourers 124.9±21.4	Rural clerks 83.4±11.6; rural labourers 80.3±11.6
26a	Male rural farmers (70)	63.0±12.6	13.9		13.9	2.8		2.8	121.1±17.9	70.6±10.9
26b	Retired railway workmen (70)	60.2±8.5	29.1		29.1	13.8		13.8	127.0±24.2	76.1±13.5
27	Market workers (40)	38.0±13.3	46.3	37.7	42.2				129.2±20.8	84.5±14.5
28	Medical school lecturers (59)^d^	46.1±9.6	24.5	15.4	21.3					
29	Musical shop operators (83)^d^	26.9±7.6			55.6				134.7±14.3	88.4±9.7
30	Pharmaceutical industry workers (46)^d^		51.3	43.5	48.0					
31	Policemen and male civil servants (52)^d^	Policemen 23.2 years; civil servants 28.6 years	28.7		28.7	8.5		8.5	Policemen 130.0±15.3; civil servants 134.0±18.8	Policemen 76.0±14.4; civil servants 75.0±14.9
32	Port workers (76)	46.0±6.0	32.6	11.1	29.7					
33	Professional drivers (41)^d^	41.1±6.1	22.5		22.5					
34	Rubber plantation workers (43)				12.5				Men 125.6; women 123.0	Men 72.4; women 71.9
35	Senior executives of industries and companies (68)^d^		36.1	29.8	34.7	5.8	12.8	7.4	Men 129.8; women 127.4	Men 82.6; women 80.1
36	Staff of a government organisation and private factory (67)^d^	40.3±9.6	28.4	22.9	27.0				130.4±20.5	83.3±12.0
37	Traders (38)^d^	43.9±12.7	21.2	43.4	36.3	8.2	18.1	15.0	128.9±23.5	80.5±12.7
38	Traditional chiefs (77)^d^	56.5±4.1	68.9		68.9					
39	University staff (60)	40.5±10.8	40.0	21.7	34.0					
40	University staff (57)^d^				29.0					
41	University staff (58)^f^	37.4±9.5			21.5					
42	University staff (61)	Men=41; women=43	11.2	17.3	13.4					
43	Vegetable, cereal and tuber mill operators (72)	41	20.0	18.2	19.2					
44	Information technology workers (31)	46.2±7.6	26.3	20.6	24.1	14.3	7.4	11.9		
45	Telecommunication workers (69)	41.8±9.1			43.7				151.7±13.6	97.1±9.1

a Based on SHT

b based on DHT

c mean BP: Addo et al. (30) uses median SBP and DBP

d definition of HTN: based on measurement only

e BP threshold 160/100 mmHg

f based on self-reported previous diagnosis of HTN. BP=blood pressure; HTN=hypertension; SBP=systolic blood pressure; DBP=diastolic blood pressure; SHT=systolic hypertension; DHT=diastolic hypertension.

Young age did not automatically mean lower prevalence of hypertension. Among music shop operators in Benin City, Edo State, Nigeria, with a mean age 26.9±7.6 years, 55% were hypertensive (83). Most of the shop operators were exposed to high noise levels >90 dB for more than 8 h daily.

Of the 40 studies, the prevalence of hypertension in workers was ≥20% in 30 studies (15.0%), ≥30% in 15 studies (37.5%), and ≥40% in 6 studies (15.0%) (Table 3). The prevalence was high among both formal and informal sector workers. A slightly higher proportion of studies among informal sector workers reported a prevalence ≥30% than those among formal sector workers (6/15 vs. 9/23 studies). High prevalence (≥30%) informal sector workers included traders (38, 39), market workers (86), music shop operators, and chiefs (77). Among formal sector workers, they included civil servants (30, 48, 56), health workers (62, 80), university staff (60), port workers (76), factory workers (46), senior executives (68), and telecommunications workers (69). There were variations in the prevalence of hypertension within a particular group of workers. For example, the prevalence was 17.5% among health workers in Abidjan, Cote d'Ivoire (64), but was twice as much among health workers in Jos City (36.5%) (62) or Umuahia, Nigeria (37.5%) (80). Similarly, the prevalence among civil servants in Accra in 2006 (30) or Bendel State in 1988 (48) was twice that among civil servants in the Bendel State in 1992 (51) (30% vs. 16%).

Among the informal sector workers, the prevalence of hypertension was lower among jobs that were more physically demanding such as automobile workers (12.0%) (71), industrial workers (12.3%) (42), plantation workers (12.5%) (43), and mill operators (19.2%) (72) and plantation workers (12.5%) (43). However, these workers also tended to be younger. In contrast, the prevalence was generally higher among largely sedentary workers such as traders (39, 86), office executives (68, 69), and civil servants (30, 48, 56).

In 10 studies that used the higher cut-off point of 160/95 mmHg, the prevalence of hypertension ranged from 5.9% in Sokoto civil servants (79) to 16.3% among Benin City civil servants in 1987/1988 (47, 48). Among men, the prevalence of hypertension ranged from 2.8% in rural farmers to 17.8% among Benin City civil servants (Table 3). Among women, the prevalence ranged from 3.5% among civil servants and factory workers in Calabar (50) to 12.8% among senior executives in Benin City (68).

In 26 studies with available data, the prevalence of hypertension based on the 140/90 mmHg cut-off point in male workers ranged from 11.2 to 68.9% while in the 22 studies with available data, it ranged from 9.9 to 43.5% among female workers (Table 3). It was consistently higher in males than in females in 16 studies. The differences were not insignificant – in 12 cases, the prevalence was more than five percentage points higher in males. The greatest sex differences of 18–21% were observed in three studies – among university workers at the University of Ghana, Accra (60), among civil servants in the Bendel State in Nigeria (48), and workers at the Port of Abidjan, Cote d'Ivoire (76). In these three studies, the prevalence of hypertension among males was about two to three times that among women. In the other three studies in which there was a reversal, the prevalence of hypertension in females was 1.2–2.0 times that in male and the percentage points difference ranged from 5.0 to 22.2% (38, 55, 61).

The sex differences in the prevalence of hypertension were less pronounced but also mostly higher in male workers than female workers when the BP cut-off point of 160/95 mmHg was used. Five studies reported a higher prevalence among males (48, 50, 53, 73, 79) with a 2.1–8.7% points difference while two studies reported higher prevalence among females (45, 68).

Almost all the studies with available data consistently showed a direct relationship between age and mean systolic and diastolic BP and hypertension irrespective of sex or geographical setting (Table 4) (37, 74, 80, 87). In the few studies conducted in mixed settings, the mean SBP, mean DBP, and the prevalence of hypertension were higher in urban than in rural areas (70, 82). The prevalence of hypertension was higher among senior staff than among junior staff (30, 57, 60, 88), even after adjusting for age (48, 79). For example, the age-adjusted prevalence in senior and junior staff was 43% vs. 23%, respectively, among male civil servants in Benin City (48). In multivariate analyses, the major determinants of hypertension included older age group, male sex, and higher socio-economic status (30, 44, 48, 53, 62, 79).

**Table 4:** Age-specific prevalence of hypertension among workers in West Africa

		15–24			25–34			35–44			45–54			55–64		
					
Study population	Location	M	F	T	M	F	T	M	F	T	M	F	T	M	F	T
Civil servants (30)	Accra						9.8			20.1			43.9			49.4
Civil servants (48)	Bendel State				20.3	12.9	18.2	36.5	16.0	32.0	52.0	44.4	51.4			
Civil servants (79)	Sokoto	8.9	3.8		12.1	17.6		23.0	23.1		54.5					
Civil servants (50)	Calabar			1.4			5.9			12.6			22.3			27.8
Civil servants (51)	Benin City, Edo State						3.9			11.0			27.1			42.5
Male civil servants (52)	Benin City, Bendel State	29.3			27.7			38.5			68.9					
Policemen (52)	Benin City, Bendel State	15.7			21.6			31.8			58.8					
University staff (58)	Ibadan															
Bank workers (73)	Ibadan	17.9	2.8	10.7	15.1	7.9	12.6	21.9	26.5	23.3	50.7	28.0	44.8	83.3	100.0	85.7
Health workers (80)	Umuahia, Abia State									31.1			36.3			57.9
Industrial and hotel workers (45)	Dakar	2.9	3.3	3.1	11.7	7.9	10.4	17.9	21.8	19.2	36.1	47.7	39.6			
University staff (61)	Lome	0	0		2.3	0		8.93	12.3		16.7	25.2		43.8	40	

M=males; F=females; T=total sample.

When analyses are restricted to the 32 studies with representative samples of workers, the patterns of hypertension changed very little. As before, the prevalence ranged from 12.3 to 68.9% in the 27 studies using the 140/90 mmHg BP threshold. The prevalence was ≥20% in 22 studies (81.5%), ≥30% in 10 studies (37.0%), and ≥40% in four studies (14.8%). Studies among informal sector workers were more likely to report a prevalence ≥30% than formal sector studies (5/11 vs. 5/15). The prevalence in male workers ranged from 16.0 to 68.9% while that in female workers ranged from 9.9 to 43.5%. As before, the prevalence of hypertension was most frequently higher in male workers than in female workers. The sex differences in the prevalence in these studies ranged from 2.0 to 21.5 percentage points. Only two of the 12 studies reported a higher prevalence in females (38, 55).

There was a 3.4-fold difference in the prevalence of hypertension among representative samples of civil servants (51, 84), a 1.8-fold difference among health workers (62, 65), and a 1.6-fold difference among university workers (58, 60). The differences remained when the age-specific prevalence was analysed among civil servants (Table 3). Among civil servants aged 25–34 years, the prevalence in Benin City was 3.9% (51) compared with 9.8% in Accra (30). The earlier reported heterogeneity in the characteristics of subjects, study populations, and measurement techniques of BP remained in the restricted subset of higher quality studies. The results of the entire set of retrieved studies are therefore reported.

### Trends

It was not possible to directly estimate trends in the prevalence of hypertension in workers. The Benin City Civil Servants Studies conducted in 1987–1988 and in 1992 by the same group of investigators did not have comparable study populations (48, 51). The subjects in the earlier study were from a greater number of ministries, and were more likely to be female, younger, and heavier. As expected, the prevalence of hypertension was twice as much in the earlier study (30.4%) as in the latter study (15.8%) after only a 5-year interval.

In 1977, Oviasu and Okupa screened civil servants aged 15–60 years in Benin City and found 13.3% of them had hypertension based on the 160/95 mmHg threshold (54). Ten years later, the Benin Civil Servants Study reported a prevalence of 16.3% in workers aged 25–64 years (47).

In the late 1990s, Kadiri et al. observed that the age-specific BP levels on their study among bank workers in Ibadan were lower than those of other urban workers (factory and sales workers) from their studies three to four decades earlier (1, 73). They concluded that the prevalence of hypertension appeared ‘not to have changed dramatically in the same urban workforce over the last three to four decades’ (73).

As a proxy for overall trend, the crude prevalence of hypertension based on the BP 140/90 mmHg threshold for the studies published was obtained for each decade as the quotient of the sum of hypertensive workers and the total number in the study sample in that decade (89, 90). It increased steadily from 12.9% in the 1980s, through 18.5% in the 1990s to 31.9% in the 2000s. The prevalence for studies published from 2010 to 2014 was 34.4%.

### Severity of hypertension

Evidence of the severity of hypertension is derived from the prevalence of grade 2 (moderate) hypertension, presence of target organ damage, and history of hospitalisation. Twelve to fifteen percent of health workers (62), market workers (40), traders (38), and information technology workers (31) had moderate hypertension (BP ≥160/100 mmHg). Six percent of civil servants in Accra (30) and 8.0% of market women in Enugu, Nigeria, had severe hypertension (40). Among civil servants in Accra diagnosed with hypertension, 25.4% had moderate hypertension and 19.2% had severe (Grade 3) hypertension (30). Nearly half (47.5%) of the hypertensive subjects examined had evidence of target organ damage (14). The odds of having hypertensive target organ damage was 5–6 times as much in severe hypertensives (BP ≥180/110 mmHg) as in those with BP <140/90 mmHg and increased with increasing BP.

The prevalence of hypertension among 534 apparently healthy traders who did not have diabetes in a market in Ibadan was 36.3% (38). Among those with hypertension, 41.2% had Stage 2 hypertension with BP ≥160/100 mmHg and 63.4% had metabolic syndrome.

The prevalence of left ventricular hypertrophy (LVH) ranged from 3.1 to 29.1% among 766 civil servants with a mean age of 41 years working in Benin City, Edo State, depending on the criteria used (51). It occurred 2.3–4.4 times as frequently in those with hypertension as in those with normal BPs, with prevalence of up to 48.8% among hypertensives, depending on the criteria used. The prevalence of LVH increased with increasing stage of hypertension, being 6.5–29.6 times as frequent in those with Stage 3 hypertension as in those with Stage 1 hypertension. After adjusting for age and systolic BP, body mass index and chest depth were independently associated with LVH in the male civil servants.

Among 73 traditional chiefs in oil-rich communities, 28.8% reported having ever been hospitalised as a result of the hypertension (77).

### Co-morbidity and clustering of risk factors

Workers with hypertension frequently had other cardiovascular risk factors as well as other NCDs. Among 402 Senegalese workers of information technology companies, 44.8% were diagnosed with at least one NCD (31). Of those diagnosed with NCDs, 38.9% had two NCDs and 12.2% had three NCDs. Only 15.5% had isolated hypertension while 38.3% had hypertension in combination with at least one of three diseases – chronic kidney disease, obesity, or diabetes.

Nearly one-fifth (18%) of telecommunications workers in Senegal diagnosed with hypertension were estimated to be at high risk of a cardiovascular event, based on the Framingham risk score (69). The major cardiovascular risks were hypertension (43.7%), physical inactivity (68.0%), hypercholesterolaemia (37.9%), LVH (17.0%), obesity (11.3%), and tobacco use (12.3%). Twenty-eight percent of the workers had one risk factor, 51% had two risk factors, and 21% had three or more risk factors.

Ige et al. observed that NCDs and high-risk behaviour were common among university staff in Ibadan (58). Ninety-six percent of the staff reported unhealthy diets, 27% low physical activity, 5% excessive alcohol intake, and tobacco smoking 2%. While 67.4% reported only one risk behaviour, 29.9% reported multiple risk behaviour. Overall, 21.5% of the university workers reported having been diagnosed with hypertension and 11.1% with diabetes.

Among 52 oil workers in the Niger Delta region of Nigeria diagnosed with diabetes, 38.5% were obese, 46% were hypertensive, 67.3% had metabolic syndrome, and 88.5% had dyslipidaemia (91). In Lagos, 22.3% of hypertensive bank workers had diabetes compared with 1.9% of non-hypertensives (74). Of the bank workers with diabetes, 75.8% had hypertension compared with 17.5% of non-diabetics.

### Awareness, treatment, and control

Among the various workers with hypertension, 20–84% were aware of their hypertensive status (Table 5). As expected, health workers and the most highly educated group of workers were most likely to be aware of their hypertensive status. Specifically, 65–84% of hospital workers (62, 64) and 75% of medical school lecturers (59) previously knew they were hypertensives. In contrast, only 29% of market workers comprising traders and artisans knew they had hypertension (40). Beyond these groups of workers, the patterns of awareness were variable. For example, 73.9% of mill operators in an Ibadan market (72) compared with 22–24% of civil servants in Lome (56) and Ibadan (53) were aware of their previous diagnosis of hypertension.

**Table 5:** Awareness, treatment, and control of hypertension among workers

		No. of hypertensives BP≥140/90	No. of hypertensives BP≥160/95^a^	Among all hypertensives %			Treatment among those aware %	Control among those on treatment %
	
Study population	Location			Awareness %	Treatment %	Control %		
Bank workers (73)	Ibadan	264	85	49.4				
Civil servants (53)	Ibadan		103	24.3				
Civil servants (30)	Accra	307		54.1	31.3	12.7	82.8	40.6
Civil servants (56)	Lome	112		22.3	1.8	1.0	8.0	100
Civil servants (51)	Benin City	121				8.3		
Civil servants, factory and plantation workers (50)	Calabar		419	19.6				
Factory and hotel workers (45)	Dakar		152	64.9	24.5	12.6	37.8	51.4
Health workers (62)	Jos City	124		66.1	43.5		65.9	
Health workers (64)	Abidjan	144		84.0	79.2		94.2	
Information technology workers (31)	Dakar	97		68.0				
Market workers (40)	Enugu	290		29.3				
Medical school lecturers (59)	Port Harcourt	16		75.0	75.0		100.0	
Mill operators (72)	Ibadan	23		73.9				
Policemen and male civil servants (52)	Benin City		95		9.7			
Rubber plantation workers (43)	Rural	448			0			
Telecommunication workers (69)	Not stated	537			14.9			
Traditional chiefs (77)	Rivers State	73		54.8	50.7		80.4	
University staff (60)	Accra	48		43.8	43.8	0.0	100.0	0

a % hypertensives aware of their status or on treatment is based on BP 160/95 mmHg cut-off.

On the whole, awareness of hypertension status was high except in four studies in which only about one-fifth to half of civil servants, plantation and factory workers (50), civil servants (53), university workers in the health sciences departments (60), and bank workers (73) knew about their hypertension. There were still important awareness gaps among workers. Even in the study reporting the highest rate of awareness in Abidjan, 15% of the doctors with hypertension were newly diagnosed (64).

The proportion of workers with hypertension who were on treatment ranged from 0% among rubber plantation workers to 79% among health workers. Again, health workers and medical school lecturers were most likely to be on treatment. In nearly all of the studies with available data, less than half of workers with hypertension were on treatment. Among the traditional chiefs from oil-rich communities in Nigeria in whom the highest prevalence of hypertension was observed, 55% were previously aware they had hypertension and about half were on medication.

Treatment rates improved dramatically to 38–100% when assessed among those hypertensives who were previously aware of their diagnosis. Most workers including medical staff on treatment were on monotherapy (62, 64). In the five studies in this review in which it was reported, the controlled rates ranged from 0 to 13% among all hypertensives and from 0 to 100% among those on treatment.

### Knowledge and perceptions

Knowledge about the aetiology and management of hypertension among some workers is inadequate. Seventy-three percent of university workers in Nigeria thought hypertension was caused by undue thinking, stress, or worries, and 65% did not know it required life-long treatment (92). Among hospital workers in Abeokuta, Nigeria, 89% correctly identified hypertension as a risk factor for stroke while 15% attributed it to evil spirits or the will of God (93). However, 29% of the workers (of whom nearly a quarter were clinical workers) could not identify the brain as the organ affected. While 61% preferred hospital treatment, 13% preferred spiritual treatment. Higher level of education and being a clinical worker were significantly associated with adequate knowledge of stroke.

Similarly, senior and junior staff of the University of Calabar, Nigeria, had poor knowledge of the risk factors for ischaemic heart disease (94). Only 6–42% knew obesity, sedentary lifestyle, and oral contraceptives were risk factors for ischaemic heart disease. Forty percent considered hypertension as a leading NCD cause of death; even less knew about heart attack, diabetes, or cancer as leading causes of deaths.

There was sometimes a disconnection between the perception and the reality of being overweight or obese. Whereas 72% of health workers in a university teaching hospital were found to be overweight or obese, only 27% perceived themselves to be overweight (62).

### Compliance

There were scant reports of non-compliance with treatment among workers in West Africa. Poor compliance was observed even among health workers. In Abidjan, 71% of health workers, particularly assistant nurses and nurses, had difficulties complying with their antihypertensive treatment (64). Contrary to expectation, persons on multiple therapy were more compliant than those on monotherapy.

In Lome, 92% of all hypertensive civil servants who were aware of their diagnoses had stopped taking their medication or would only take them when they had symptoms (56). Sixty percent of the hypertensives had been first diagnosed during the course of an illness.

### Uptake of medical check-up

Consistent with their low awareness and knowledge about CVDs, West African workers infrequently underwent a medical check-up. Hypertensive senior executives in Nigeria had not had a check-up for a varying period of 0–22 years with a mean of 2.8±1.7 years (68). Among university workers in Calabar, only 25% had visited the hospital for a routine medical check-up (94). Most only went to the hospital when they were ill.

Similarly, Funke and Ibrahim found that most (58.5%) health workers in the Jos University Teaching Hospital of Nigeria rarely checked their BP except when they were ill (62). Thirty-five percent had not checked their BP in the preceding 1 year. Medical staff, senior staff, and those with tertiary education were more likely to regularly monitor their BP. More than 60% rarely checked their weight or had never done so.

## Discussion

This is the first review of the prevalence of hypertension as well as its awareness, detection, and control among workers in West Africa. A particular strength of this review is the identification of a large number of studies including those in which the prevalence of hypertension was a secondary or an incidental objective. The review also identified some publications in French, which are usually not included in English-language-based reviews. It covered a wide range of formal and informal sector workers. Of particular interest is the behaviour of health workers who are expected to be models of healthy living in their societies.

However, it is possible that the review under-represented studies among workers in French- and Portuguese-speaking West African countries. The search in French was only limited to Google Scholar and fewer French or Portuguese journals are included in the databases that were searched. There were two publications from French-speaking Senegal that were published in English (31, 45). The findings among the different workers may not be generalised to the general population in West Africa. However, many of the findings, such as the high prevalence of hypertension, low awareness, and control, reflect patterns in the general population (15, 89, 95). Hypertension is now a pandemic in West Africa with significant levels, not only in the urban populations, but also rural populations (96) and marginalised groups (97). Locating articles was particularly difficult as several publications, particularly older journals, and the most recent publications of African journals were not available online. Comparisons of the prevalence of hypertension between workers from different studies were difficult due to the wide diversity of the groups and the lack of standardised rates. However, age-specific prevalence rates were obtained where available. There were differences in the methods of measurements of BP in terms of type of equipment, number of measurements, number of visits, and in the values analysed to compute the final BP.

Assessing the quality of studies was complicated by the methodological challenges in data collection and reporting deficiencies. Most of the studies enrolled a representative sample of workers. Staff lists were available for most formal sector workers as well as for some informal sector workers. Studies without staff lists strived to achieve high participation through active door-to-door mobilisation of available staff or the cooperation of the workers’ leadership. Non-participation may have the effect of biasing the prevalence in either direction. However, as has been rightly argued, any effect is likely to be minimal owing to the prevailing low awareness of hypertension, even among these formal sector workers (79).

Several important findings emerged from the review. Only a few of the ECOWAS Member States have published any studies on hypertension in workers. Nigeria alone produced the overwhelming majority of all the identified studies, likely a reflection of its large population and research capacity. The production rate of studies has dramatically increased over the past 5 years compared to 20 or 30 years previously. The strikingly low production of studies and systematic reviews on NCDs from low-income settings has been described (98, 99). Low nutrition capacity has also been recently described in West Africa where only nine countries offer any degree programmes (100).

The review showed high prevalence of hypertension particularly among the men, older group, and senior staff. The prevalence of hypertension in male civil servants in Nigeria was reported to be similar to that of US black males (48). High prevalence of hypertension of up to 50.2% among men and 68.8% among women was similarly reported in a 60-year review of hypertension in Nigeria (89). In Ghana, a systematic review reported a prevalence of 19.2–48.0% (95). Addo et al. observed lower prevalence of 12.5–29.4% in their systematic review of studies in Africa (15). Studies which excluded persons with diabetes or known hypertension from their sample (65, 71) or only measured systolic hypertension may have underestimated the true prevalence in the workers.

In contrast with the findings of national (95) and regional reviews (15, 101) which reported minimal sex differences, this review found higher prevalence among male workers in most studies. It is not clear if sex difference reflects the differences in the study populations or methods. Consistent with this review, most STEPS surveys in Africa and other studies have found a higher prevalence of hypertension among men (16, 101). It has been proposed that the molecular mechanisms underlying vasculature, nervous system, and kidney functions that lead to hypertension and the pathways for the control of BP may explain the differences between the sexes (102).

The review also highlighted high levels of hypertension among sedentary groups such as traders, and traditional chiefs or the informal sector who are rarely targeted by national cardiovascular health programmes or policies. Models for workplace or employee well-being programmes are almost exclusively based on structured formal sector workplaces (103). In low-income settings, it is important for national programmes on occupational health and NCDs to target these informal sector workers through their unions and associations and integrate these programmes into their work-related activities (39).

Severe hypertension and target organ damage were relatively common among those with hypertension. This was probably caused or worsened by the prevailing low awareness, treatment, and poor control rates, infrequent medical check-up, co-morbidity, low compliance with treatment, inappropriate therapy, and use of plant medicine with unproven efficacy (104). In West Africa, many hypertensives are diagnosed for the first time when they present with complications such as stroke or heart failure (105, 106). In one tertiary hospital in Nigeria, for instance, about half of the hypertensive-related admissions were undiagnosed (107). Autopsy findings also confirm sudden deaths from undiagnosed hypertension (108). Co-morbidity and clustering of risk factors, which was relatively common among workers, have also been reported among the general adult population (86). Contrary to the observation of Kadiri and his colleagues 15 years ago (73), there is evidence from a systematic review of increasing prevalence of hypertension in Nigeria (109). Evidence of increasing trend is supported by another systematic review in Nigeria which found that the crude decade prevalence increased from 8.6% in 1970–1979 to 22.5% in 2000–2009 (89).

As they are better educated and are more likely to undergo medical examination, workers were more likely to be aware of their hypertension than the general population (110). Improving access to screening programmes through community-based health insurance schemes has been shown to reduce mean population arterial BP (111, 112).

It is remarkable that high prevalence of hypertension and inadequate health behaviour were observed even among health workers, lecturers, and senior officials. Health care workers, whether or not engaged in clinical care, have the opportunity to undergo a periodic medical check-up. There is some concern that health workers with unhealthy behaviour may not be well disposed to counselling patients or providing care relating to that behaviour (113). There are some emerging initiatives. For example, the Ghana Health Service has established a gymnasium and encourages sub-national level health authorities to organise regular corporate physical activity sessions (114, 115). Ghana also has a Regenerative Health and Nutrition Programme that actively engages traditional chiefs in physical activity. Cote d'Ivoire has a website (www.preventionci.net) dedicated to creating public awareness about NCDs.

The poor control of hypertension has been confirmed in other reviews (17, 30, 95, 110). Uncontrolled systolic and diastolic BPs are a risk factor for increased cardiovascular and all-cause mortality (116). There is inadequate knowledge about treatment targets among practitioners (117). Patients are not adequately counselled about treatment and lifestyle modifications, leading many hypertensives to believe that they need to take medication only when they have symptoms (118).

Successful interventions to reduce BP in low-income countries include health education, worksite exercise breaks, training of health care staff, and introduction of guidelines (119). Workplace health promotion interventions have been beneficial in improving workers’ physical activity, dietary behaviour, and weight (120). The West African Health Organisation (WAHO) has the mandate to support countries to implement these interventions. In line with this mandate, WAHO has supported several ECOWAS Member States to develop or revise their integrated NCD strategic plans and policies, conduct STEPS risk factor surveys, and strengthen NCD care at the primary care level. In 2013, WAHO in collaboration with WHO published a consensus statement on dietary salt reduction (121). As WAHO prepares to develop a regional NCD strategic plan, it now has the opportunity to give more priority to the control of NCDs in workplace settings.

## Conclusions

The prevalence of hypertension among workers in ECOWAS Member States is quite high. A significant proportion of the disease is undiagnosed, severe, and complicated. While better than that of the general population, the awareness, treatment, and control of the disease is low. Workers have little knowledge of the disease, and they infrequently undergo a medical check-up. Occupational health programs should aim to improve the general awareness of workers, promote healthy behaviour, screen for risk factors, and institute integrated control of NCDs.

## Author's information

WKB works as the Professional Officer in charge of nutrition and non-communicable diseases at the West African Health Organisation.
